# Quality Assurance in Archaeological Survey

**DOI:** 10.1007/s10816-016-9274-2

**Published:** 2016-02-12

**Authors:** E. B. Banning, Alicia L. Hawkins, S. T. Stewart, P. Hitchings, S. Edwards

**Affiliations:** 1 Department of Anthropology, University of Toronto, 19 Russell St., Toronto, ON M5S 2S2 Canada; 2 Archaeology Program, School of the Environment, Laurentian University, 935 Ramsey Lake Rd., Sudbury, ON P3E 2C6 Canada; 3 Trent University Archaeological Research Centre, Trent University, 1600 West Bank Dr., Peterborough, ON K9J 7B8 Canada; 4 Department of Near and Middle Eastern Civilizations, University of Toronto, 4 Bancroft Ave., Toronto, ON M5S 1C1 Canada

**Keywords:** Archaeological survey, Fieldwalking, Quality Assurance, Sweep widths, Coverage

## Abstract

To have confidence in the results of an archaeological survey, whether for heritage management or research objectives, we must have some assurance that the survey was carried out to a reasonably high standard. This paper discusses the use of Quality Assurance (QA) approaches and empirical methods for estimating surveys’ effectiveness at discovering archaeological artifacts as a means for ensuring quality standards. We illustrate with the example of two surveys in Cyprus and Jordan in which resurvey, measurement of surveyor “sweep widths,” and realistic estimates of survey coverage allow us to evaluate explicitly the probability that the survey missed pottery or lithics, as well as to decide when survey has been thorough enough to warrant moving to another survey unit.

## Introduction

When archaeologists present the results of an archaeological survey, whether to an academic audience or to cultural resource managers, the consumers of these reports have a reasonable expectation that claims about the presence or absence of archaeological materials, about the density and distribution of materials, and about the character of the materials themselves are accurate. However, reports of many archaeological surveys include no information at all that would allow us to evaluate these claims. How effective was the survey at detecting archaeological materials? Were all field personnel adequately trained and equally effective at the detection of the full range of materials? Did the survey design take differential visibility into account? Were there tests or audits of the survey’s effectiveness and reliability? Without answers to these questions, we have no rational basis for comparing the results of different surveys or even for confidence in reports’ recommendations about development or site protection, no matter what survey methods were employed. It is possible that an area “cleared” for development might include a significant archaeological site that, for example, would qualify as significant under Section 106 of the National Historic Preservation Act (NHPA) in the United States or similar legislation elsewhere (e.g., Crombé and Verhegge 2015: 457).

Answering these and other questions about the quality of the research and data that result from it is among the concerns of Quality Assurance (QA). Some private-sector archaeological assessment companies as well as government agencies charged with their oversight have adopted QA policies, most notably the Institute of Field Archaeologists in the United Kingdom (IfA 2008a, b; see also Willems and van den Dries, eds. 2007). Others have standards and guidelines that set quality goals but do not use QA language (Register of Professional Archaeologists n.d.). Some of those that do use QA terminology, furthermore, have focused on business-management practices—that is, they have the committees and management structure in place, audit compliance with regulatory frameworks, and survey client satisfaction and employee productivity—yet in some cases have overlooked the need to ensure the quality of the product itself through design assurance and testing. For example, the Ontario Heritage Act allows appointment of inspectors (Ontario 2005), but field inspections are rare unless the Ministry receives a complaint or a problem is identified during report review, and the main emphasis of QA is to assess these reports. It appears to be rarer still for heritage assessment companies to conduct their own audits of field procedures.

## What is Quality Assurance?

Here, we cannot do justice to the more general topic of QA, so we refer readers to some of the many introductions to the topics of Quality Assurance and Quality Management (e.g., Arora 1998; Hughes and Williams 1995; Schlickman 2003; Stewart et al. 1996; Willborn 1989). In the USA, standards for quality are found in ISO9000 and ISO9001, and ANSI/ASQC Q91-87 through Q94-87, while the Canadian equivalents are CSA Z299.0 and CSA Z299.1/.4, and those in the United Kingdom are BS 4891 and BS 5750.

Briefly, QA involves policies, procedures, manuals, standards, and systems, arranged with the goal of ensuring and continually improving the quality of products or services and consumers’ satisfaction with them.

Unfortunately for archaeologists, most of the literature on Quality Assurance and Management concerns manufacturing and service industries. In the context of archaeological research, even in the private sector, we must be mindful of the fact that our product is not like television monitors or automobiles. Consequently, some aspects of QA require modification to fit our unique situation, although we do share with other research endeavors some aspects of quality research, and we also share with manufacturing businesses (especially in the case of CRM-related archaeology) the needs to monitor costs and maintain or improve the satisfaction of clients.

The purpose of QA is not to create unwieldy bureaucracy or still more unwanted paperwork; it is to put mechanisms in place so that we may consistently maintain and improve the quality of work. Undoubtedly, this creates real costs, in time, money, and training, but the failure to ensure quality also has costs, such as the need to resurvey territory that was not adequately surveyed the first time or, worse yet, the need to mitigate a site that construction crews unexpectedly uncover in an area supposedly clear of significant archaeological resources. For example, Schiffer (1987: 353) mentions a survey that failed to record a large Hohokam site in Arizona. This led to enormous and unexpected mitigation costs ahead of construction of an aqueduct that could easily have been re-routed had the survey reported the site. Yet, another cost of such failure is the psychological and social impact to descendants and the community in general when a development project inadvertently exposes an unexpected cemetery site.

In what follows, we will not attempt to grapple with the entire gamut of QA as it applies to archaeological surveys, which would have to include such topics as creating manuals and checklists, managing the quality of publications and reports, monitoring the condition of artifact collections, evaluating the effectiveness of sampling designs, and even improving the marketing of results for archaeological assessment firms. Instead, we focus on ways that the QA paradigm might apply to the design of archaeological surveys, especially by fieldwalking, the evaluation of completed surveys and the performance of field personnel, improvements to survey quality, and the mitigation of nonconformance to standards in survey. For the most part, this concerns those parts of the Quality System that deal with inspection and testing to verify that the product—in this case archaeological data—meets or exceeds expectations (ISO 9000, section 4.10–12, Paradis and Small 1996:41–49). As even this is a very broad topic, we will further focus on the important problem of survey coverage or, to put it another way, the probability that we have successfully detected the sites and artifacts that are potentially available to be found.

## Quality in Archaeological Survey

In archaeological surveys, some very important, although by no means only, benchmarks of quality are a survey’s ability to detect archaeological materials, classify them correctly, and adequately represent their character, density, and distribution. This ability in turn depends on the skill and attentiveness of field personnel, the quality of the survey design, the character of the archaeological materials, and characteristics of various “environmental” factors that affect surveyors’ attention and ability to see artifacts (Banning et al. 2006, 2011; Hawkins et al. 2003). A number of archaeologists have called attention to the importance of ensuring survey effectiveness (e.g., Hirth 1978; Ives 1982; Miller 1989; Shott 1992; Sundstrom 1993; Zubrow 1984), yet this call has had relatively little impact on practice.

## Current and Recent Practices in Archaeological Survey Quality

Many jurisdictions have standards to which consulting archaeologists must adhere when undertaking archaeological survey of lands subject to impacts by development. In the USA, the Secretary of the Interior’s Standards and Guidelines (SISG) serves as a basis that is modified or added to by individual states depending on local conditions (National Parks Service 1983). In Canada, standards are determined individually by province (e.g., Ontario Ministry of Tourism and Culture 2011). Both SISG and the Canadian systems define minimum qualifications of lead archaeologists (licensed archaeologists, permit holders, or principal investigators) and the minimum fieldwork standards to be met during survey.

The SIGS qualifications for a professional archaeologist are to ensure that individuals leading projects are able to undertake complicated projects and carry them to fruition. These and other similar standards typically require license holders to have credentials, such as a graduate degree in archaeology, and minimum field experience and sometimes expect them to belong to an archaeological organization with a code of ethics.

A number of jurisdictions have minimum requirements for field directors, but few have specific standards for the qualifications of field workers (Ontario Ministry of Tourism and Culture 2011; Georgia Council of Professional Archaeologists 2014; Tennessee Division of Archaeology 2009). Where it is mentioned, it appears that QA is the responsibility of the lead archaeologist. Minnesota, for example, requires the principal investigator to ensure that standards are met and notes that “qualified personnel” are one of the elements that contribute to quality (Anfinson 2005). In all cases, these standards are based on experience and education; none of them pertain to demonstrated skill of an individual to carry out survey effectively.

Many jurisdictions set standards for survey in terms of transect interval for pedestrian survey, acceptable amount of cover (by snow or vegetation) of the surface to be examined, test-pit interval, depth of test pits for shovel testing, and screen mesh size.

Table 1 shows that these vary widely. In some cases, states prefer that archaeologists determine methods appropriate to the specific project (e.g., Tennessee Division of Archaeology 2009), but the norm is for state guidelines to specify minimum standards. While there is some uniformity in the size of shovel tests, the maximum interval for these ranges from 5 to 30 m, and not all jurisdictions state that screening is necessary. While some state guidelines indicate that at least 75 % of a surface should be visible for pedestrian survey, others indicate that a switch should be made to test pitting when only 10 % of the surface can be seen. Transect intervals for pedestrian survey range from 5 to 30 m.

**Table 1:** Examples of standards for surveying in different American jurisdictions

Jurisdiction	Maximum interval for pedestrian survey	Visibility for pedestrian survey	Maximum interval for test pitting	Test pit size and depth	Screen mesh size specified
Georgia	30 m	25 % visibility or greater		30 cm by 30 cm to 80 cm below surface or impenetrable subsoil	
Illinois	5 m (suggested interval)	25 %	15 m	40 cm diameter to sterile subsoil	
Minnesota	5 or 10 to 15 m in areas of low potential	After a significant rainfall, test pitting to be used when visibility is less than 25 %	15 m in mid- to high-potential areas, 5–10 m in high-potential areas	30–40 cm with vertical sides	¼-inch
Mississippi	15 to 30 m			30 by 30 cm to sterile subsoil	¼-inch
Nevada	30 m	75 %			
New Hampshire	5 m	No snow cover			
New York	3–5 m	70 % visibility	15 m	30 to 50 cm diameter	
South Carolina	30 m, should be supplemented with test pits in high-probability areas	50 % visibility or greater	30 m	30 by 30 cm to 80 cm below surface or impenetrable subsoil	
Texas	30 m	30 %	3 per acre for small areas, 1 per 3 acres for larger acreage	30 cm diameter through Holocene deposits	¼-inch
Wisconsin	15 m	After a significant rainfall, test pitting to be used when visibility is less than 10 %			

Source: Anfinson (2005); Bureau of Land Management Nevada State Office (2012); Council of South Carolina Professional Archaeologists (2009); Georgia Council of Professional Archaeologists (2014); Illinois State Historical Preservation Office (n.d.); New Hampshire Division of Historical Resources (2004); New York Archaeological Council (1994); New York Archaeological Council Standards Committee (2000); Sims (2001); Texas Historical Commission (n.d.); Wisconsin Archaeological Survey (1997)

A number of states indicate that survey methods should be adapted to the specific conditions. In some cases, for example, they indicate that transect intervals should be tightened in high-probability areas (Anfinson 2005; Council of South Carolina Professional Archaeologists 2009). Standards in some jurisdictions, most notably in Europe, show recognition that geomorphological and other factors can make pedestrian survey, or even shovel testing, inadequate for site discovery and require a range of other techniques, such as coring or augering (e.g., Crombé and Verhegge 2015; Willems and Brandt 2004: 45).

## Some Factors Affecting Survey Quality

A large number of factors can affect the effectiveness of any survey.

### Survey Design and Methods

The personnel are only one element of the survey design. Other aspects include the allocation of search effort to a given area of space (usually expressed in the spacing of transects in fieldwalking or of test pits or auger locations in subsurface survey) and the selection of spaces to search, whether by formal sampling, predictive modeling, or more judgmental designs.

In current archaeological survey, the selection of transect intervals is largely arbitrary. From the QA point of view, the best that can be said is that surveys meet or somewhat exceed the requirements of state or provincial laws and guidelines for the spacing of fieldworkers. Yet, as noted above, these guidelines vary substantially, often allow intervals as high as 30 m, and, most importantly, are never based on empirical data on the relationship of transect interval to discovery probabilities of artifacts or intersection probabilities for sites. There is no reason why this should be so.

From the QA perspective, surveyors should be able to assure their clients or academic audiences that they have selected transect intervals that not only meet regulatory requirements, but will have resulted in some minimum (and reasonably high) probability of detecting the kinds of archaeological resources purportedly of interest. At the very least, survey reports should provide realistic estimates of survey coverage that take into account both imperfect detection by surveyors and the spacing of transects, augers, or test pits.

For test pitting, a number of archaeologists have emphasized that the probability that test pits will both intersect a site and detect its presence (on the basis of detected artifacts) depends on the area and spacing of test pits, the area, shape, and orientation of the site, the density of artifacts within the site, and whether the distribution of these artifacts is random or clustered (e.g., Banning 2002: 88-105; Nance 1979; 1983; Shott 1985; 1989). However, much the same issues are involved in fieldwalking as in subsurface testing.

The spacing of transects or test pits is related to the density of search effort, which some archaeologists describe as survey intensity (Plog et al. 1978: 389). More generally, we can summarize search density for fieldwalking as the amount of time or total length of transects devoted to searching each unit of space (Koopman 1980: 74; Stone 1975: 25). The relationship between search time and discovery probability is not linear, and increasing search effort exhibits diminishing returns (Banning et al. 2006). From the quality perspective, we should allocate enough effort to searching each space to meet some standard for the probability of detecting targets of interest, yet we should also not squander search effort on one space that could be more economically used in another one. That is, additional search effort in the first space might only increase discovery probabilities by 5 or 6 %, because the search has reached a plateau in the detection function, while the same amount of search effort in a new space might yield a discovery probability of 50 or 60 %. Since the total time available for survey is often limited (a “fixed-budget survey”), the allocation of search time has important implications and is arguably even more important for relatively costly test pitting than for fieldwalking.

Somewhat analogous to transect or test-pit spacing, the mesh size of screens and amount of time devoted to screening sediments from subsurface testing have a nonlinear relationship to the probability of detecting artifacts in the sediment (Crombé and Verhegge 2015). This is an issue that deserves attention in a separate paper, but at the very least QA requires us to report on the quality characteristics of screening in some detail.

A survey design’s plan for selecting areas to be searched, including, where relevant, the rationale for sampling, is a critical aspect of survey quality. For sample surveys, it is necessary to provide justification for the sample size and assurance that the correct formulae for statistics have been used (especially for cluster and stratified sampling, Orton 2000: 99, 211–213) and, for stratified samples, the rationale for stratification and a post-survey evaluation of the stratification’s effectiveness (i.e., are there statistically significant differences between the strata?).

### Personnel

There are good reasons to expect that crew members’ training, experience, motivation, health, attentiveness, and ability to see and recognize small objects on a varying background of soil surfaces and vegetation will vary over time and among individuals (Plog et al. 1978: 413–15). However, implicit in the reports of typical archaeological surveys is the assumption that all field crews had closely similar or identical detection abilities, some even suggesting, implicitly or explicitly, that within some specified range they detected 100 % of the artifacts exposed on the surface (this is the definite detection model; Koopman 1980:59; Banning 2002:57–59). We suggest that this assumption is, to say the least, over-optimistic, and our field experiments have shown that surveyors’ detection of artifacts, even under rather ideal conditions, is both variable and far from perfect (Banning et al. 2006, 2011; Hawkins et al. 2003).

If we are to take the results of surveys seriously, especially in their claims for the absence of archaeological remains, we should have some assurance that they have taken surveyors’ detection abilities into account. Such assurance requires both training of crew members and periodic assessment of their detection abilities under a variety of conditions.

### Artifact Obtrusiveness

The characteristics of the artifacts we are trying to detect also affect our ability to detect them. For several decades, archaeologists have summarized this effect as obtrusiveness (Schiffer et al. 1978: 6). Research by us (Banning et al. 2006, 2011) and others (e.g., Schon 2002; Wandsnider and Camilli 1992) has confirmed the intuitive expectation that, holding other conditions constant, the size, shape, color, and reflectivity of artifacts, especially as these contrast with their environment, create substantial variation in their detectability. For example, fairly bright and glossy sherds of glazed porcelain or glass are quite easy to detect on a variety of backgrounds, while mottled grey flakes of chert are quite difficult to see, especially on stony backgrounds.

Since different kinds of archaeological resources vary in the kinds and diversity of remains that signal their presence, and these remains themselves vary in detectability, we should expect claims about the density and distribution of different kinds of archaeological sites to take artifact obtrusiveness into account. For example, we should be wary of claims for a particular ratio of Early Woodland sites to historic ones in the American Northeast if these claims did not take into account that the kinds of artifacts expected on an Early Woodland site are quite unobtrusive, while those on historic sites can include bright porcelain, reflective glass fragments, and even chunks of brick.

In addition, aside from the obtrusiveness of individual artifacts, their distribution can also have an impact on their probability of detection (Krakker et al. 1983). In some cases, higher degrees of artifact clustering can result in greater artifact detection than in cases where the same kinds of artifacts are distributed randomly or relatively evenly. Since archaeologists are often interested in discovering precisely these highly clustered manifestations of artifacts, this can be advantageous. However, we also need to be aware of the potential bias that can result when archaeological resources that are more likely to exhibit high degrees of clustering are over-represented relative to less clustered remains. The tendency for surveyors to pay closer attention once they find even one artifact has the potential to exacerbate this bias.

### Visibility

In addition, variations in surface texture and color, vegetation cover, lighting, rainfall, plowing, and other environmental conditions can have profound impacts on the detection even of artifacts that are exposed at the surface, not to mention those that may be buried to various depths by a number of geological and cultural processes (Schiffer et al. 1978: 6; Banning 2002: 46–48; Fanning and Holdaway 2004; Stark and Geraty 2008). Assurance of the quality of an archaeological survey and its reports requires analysis of how these factors may have impeded artifact detection and their potential impacts on the apparent distributions of archaeological materials. A number of archaeological studies have emphasized the effects of rainfall (Shott 1995; Shott et al. 2002) and plowing (e.g., Ammerman 1985; Clark and Schofield 1997). As noted above, some jurisdictions’ standards and guidelines for archaeological survey do require reporting of fieldwork conditions, rainfall, vegetation cover, and other factors that affect visibility, yet subsequent research and predictive models that employ the data from surveys do not always take these factors into account.

## Evaluation

An extremely important aspect of archaeological survey, too often overlooked, is the evaluation of its effectiveness. That evaluation is a critical aspect of QA and should apply to all of the aspects discussed in the last section. Since space is limited, here we focus on sweep width and coverage as the best way to summarize the interacting factors of surveyor abilities, visibility, and artifact obtrusiveness.

## Evaluating Survey Effectiveness

Some survey practictioners have attempted to evaluate shortcomings in surveys by reference to one or two of the factors that can affect detection, most usually focusing on visibility in pedestrian survey (e.g., Stark and Geraty 2008; Terrenato 2000), while others focus on inter-surveyor differences, especially for neighboring surveyors in parallel transects (e.g., Hawkins et al. 2003; cf. Gnaden and Holdaway 2000). But the quality of a survey depends on all these characteristics—survey personnel, artifacts, and environment—and they interact with one another. Consequently, it is most practical to summarize these effects simultaneously. Two basic ways to accomplish this are resurvey and a calibration approach. These are applicable to any survey method, but here we focus particularly on pedestrian survey or “fieldwalking.”

## Testing of Survey Quality by Resurvey

Any QA program requires periodic tests and audits to ensure compliance with standards, assess the effectiveness of designs and reliability of results, and provide data with which to improve those results. Typically, such tests are carried out by specially trained inspectors, who may observe people at work, make measurements on and statistical analyses of samples of their output, or both.

One of the most obvious ways to test the effectiveness of archaeological surveys is an example of this approach and involves resurveying a sample of the spaces that have already been subject to survey. A number of survey projects have employed this strategy. An early example of this was the 1977–78 East Hampshire Survey, which detected marked differences in results when resurveying a sample of four fields (Shennan 1985: 44). Hirth (1978) used survey of the same spaces in three different seasons to evaluate the effects of rainfall and agricultural practices on the detection of artifacts in the Basin of Mexico. Wandsnider and colleagues used resurvey as one component in their evaluation of surveys in the Green River Basin, Wyoming, the Mesilla Bolson, and the Sandilla Mountains, both in New Mexico (Wandsnider and Camilli 1992; Wandsnider and Ebert 1986).

In an example that one of us undertook in Cyprus in the 1980s and 1990s, there were also selective examples of resurvey to test survey effectiveness (Stewart 2006). To check on the accuracy of site and find-spot locations, and to assess changing site and artifact visibility in the field, the team resurveyed a number of units from two survey projects (Stewart 2006:85–86, 103–104, 106, 111, 146–147; Stewart and Morden 2016). Initial analysis of the overall numbers of artifacts within a survey unit or across the study area suggests no differences between the original and second surveys. However, when we consider the location of finds within the area, it turns out that findspots from the resurvey were never in the same location as those from the initial survey. Although the crews collected some artifacts on the first survey, they did not attempt to collect all artifacts so, theoretically, the findspots located on the first survey should still have been detectable on the resurvey. The mostly likely explanation for this marked difference is the survey’s use of sinusoidal, rather than straight, transects, which made it impossible for the resurvey to duplicate exactly the pathways of the initial survey.

Broadly speaking, Burger et al.’s (2004; 2006) “nested-intensity sampling” is also an example of the resurvey approach. Using a variation of the Whittaker multi-scale sampling plot, they resurvey a sample of spaces at increasingly smaller resolutions and intensities, including even crawling on hands and knees, and excavation and screening of the top 10 cm of small spaces. Comparison of the results at these different levels of intensity allows estimates of how much the less intensive survey has missed.

In a particularly interesting case, Almagro-Gorbea et al. (1996; 2002) attempted to ensure reliability of survey in the Comunidad de Madrid by using an independent “control survey” of a sample of the areas that had already been surveyed. Originally, this was a “blind but directed” sample (Almagro et al. 1996:252), but the authors later realized that, for the quality control itself to be valid, it needed to be a random sample (Almagro et al. 2002: 47). In its final form, their method involved comparing the initial and “control” survey statistically, in terms of their classification of survey spaces as “sites” and “nonsites,” using an estimate of the actual number of sites and nonsites, and thus really evaluates the degree of agreement between the two surveys. This project comes closest among those we have seen to the formal principles of QA and involved use of a military standard for inspection sampling to decide how many units required resurvey. From the quality perspective, the survey-resurvey approach works best when the resurvey is always by the same, highly experienced, and highly skilled team, whose members were not involved in the original survey.

In an attempt to check on the reliability of surface collections from sites, Shott et al. (2002) re-collected an Oneota site in Iowa that had already been surveyed the previous year and compared the results. They concluded that the materials collected during resurvey differed significantly from those from the original survey and attributed a good deal of the difference to rainfall. As they note in the paper, “archaeologists rarely allow for the effects of rainfall amount when interpreting survey results” (Shott et al. 2002: 180).

While the survey-resurvey strategy is clearly much better than no testing at all, it does have several disadvantages. First, the resurvey is not always by a team of experts, and not even always by the same team. Consequently, we cannot be certain that the second survey is any more reliable or accurate than the first, and it may not be justified to take comfort even in a high degree of agreement between the two surveys. Second, even if the resurvey is by our best, most reliable surveyors, it is not plausible to assume that their results will be perfect. Indeed, our experiments have shown that even highly skilled and experienced surveyors only detect a fraction of the artifacts exposed on the surface under typical conditions. Third, since the resurvey strategy usually operates in the absence of knowledge of the *actual* distribution of artifacts, we cannot assess the real detection rates of any of the surveyors, including the most skilled ones, but can only assess their relative abilities. Fourth, the resurvey strategy is highly vulnerable to other threats to validity. It is an example of the test-retest research design and thus can suffer from the effects of history and maturation (Campbell and Stanley 1963: 5). That is, ground conditions, weather conditions, or some other relevant factor could have changed during the interval between the first and second surveys, making them not strictly comparable. This is especially likely in the event that the first survey removed significant numbers of artifacts from the surface, when surveys were in different seasons (Hirth 1978) or, as Shott et al. (2002) point out, when there were significant differences in rainfall.

Only a few examples of archaeological re-survey have employed controls that help mitigate some of these differences. One method for control is for the initial surveyors to seed survey areas with a known number of artifacts that are similar in some respects to the target artifacts, yet clearly identifiable as nonarchaeological (Wandsnider and Camilli 1992: 173–76). The proportion of these that the resurvey team discovers serves as a means to estimate their survey effectiveness, rather than just assuming that the resurvey team is perfect.

## Measuring Survey Effectiveness with Sweep Widths

Our preferred methods for assessing and improving the quality of archaeological surveys depend on tests of surveyors’ abilities to detect a variety of artifact types under a variety of controlled but realistic field conditions. To ensure that we know in advance what the population of artifacts is like, we carry out this assessment on a series of test fields, which we have seeded with a variety of artifacts in known locations (cf. Schon 2002; Wandsnider and Camilli 1992). To control for maturation and history, we assess a group of surveyors repeatedly over the course of a survey project to produce “average” results. To control for the effects of range, we measure the variation in artifact recovery at different distances from the transect lines. To control for search speed, we ask surveyors to walk at a speed that mirrors their typical speed on “real” survey.

Effective sweep width (W) is the single most useful measure of survey effectiveness because it summarizes all of the variables that affect survey detection in a single number, with the proviso that we either need to measure it separately for each artifact type or make some assumptions about the likely mixture of artifact types. Where possible, it is better to take the former approach, since we rarely have much *a priori* information about the likely proportions of artifacts to expect. In the example below, however, we compromise by grouping artifacts into two broad categories, lithics and pottery, and omit very small artifacts, such as microliths, that are very difficult to detect. One reason for this is that it improves our sample size, as we have found that our data sets for individual artifact classes are sometimes too sparse for really good estimates of sweep width.

**Fig. 1:**
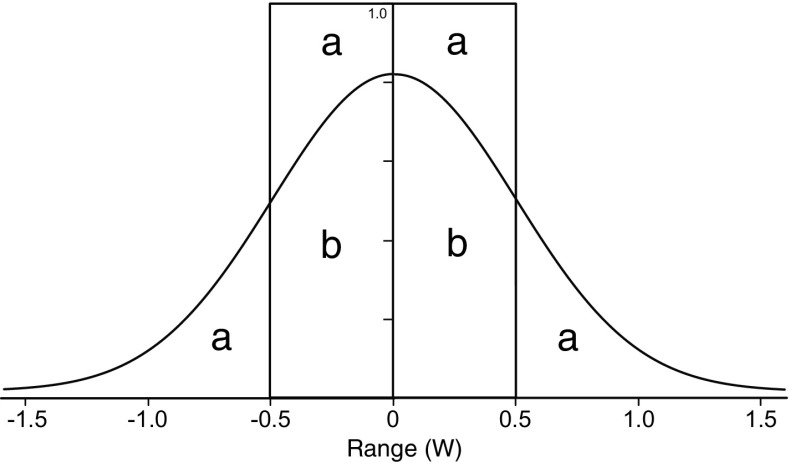
Detection functions and the definition of sweep width (W). The *rectangle* defines the definite detection model, with perfect detection within W/2 of the transect line. With a width of W m and a height of 1.0 (with no units), the area of this *box* is W m = 2(*a* + *b*). The *curve* is an example of a more realistic exponential model, with 2*b* detections within W/2 and 2*a* detections outside that range, in the tails. Consequently, the area under the *curve* is also 2(*a* + *b*) = W m

On the basis of data from controlled calibration transects, described below, we can calculate sweep widths from the exponential detection function

1 $$ p(r)=b{e}^{-k{r}^2} $$

where *p(r)* is the probability of detection at range *r* in meters, *b* is the y-intercept (expected detection probability right on the transect, where *r* = 0), *e* is the exponential constant (approximately 2.718), *k* is a constant that summarizes the effects of the various contributors to detectability per square meter, *r* is the range or perpendicular distance away from the transect line in meters, and *kr* ^2^ describes the steepness of falloff in detectability away from that line (Koopman 1980: 64; Banning et al. 2011: 3448). This function typically describes an S-shaped curve of declining probability away from the transect line (Fig. 1).

**Fig. 2:**
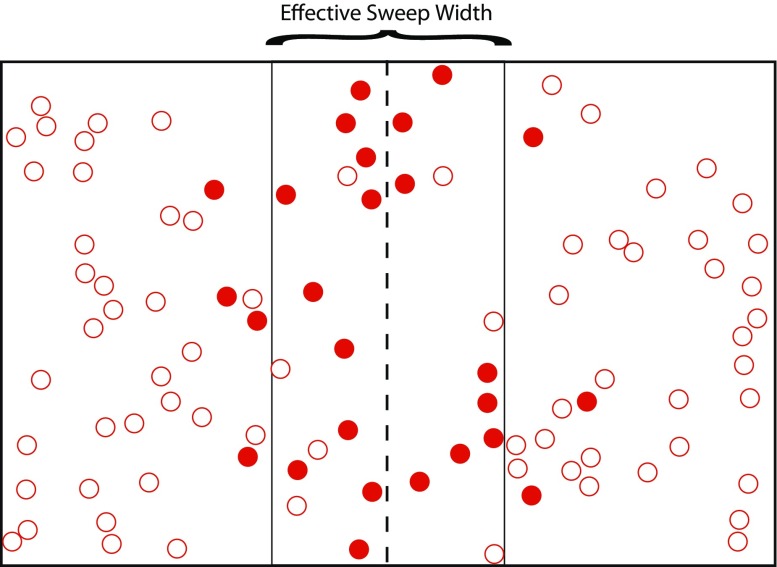
A map view of the definition of sweep width. *Filled circles* represent successful detections, *open circles* undetected artifacts. Note that the number of *open circles* within W/2 equals the number of *closed circles* outside W/2 (after Robe and Frost 2002: 10)

Sweep width in meters is equal to the area under this curve (or double the area if we only consider positive values of range). The reason that this area is in meters (not square meters) is that the y-value is a probability, with no units at all. This is clear from the fact that the sweep width corresponds with a box (Fig. 1) whose height is 1.0 (the probability), whose width is W (in meters), and whose area, in the figure, is 2(*a* + *b*). This box defines what is known as the definite detection model (Banning et al. 2006), whereby we expect to find all the artifacts within W/2 of the transect line, but none at all beyond that range. In the more realistic exponential model described by the curve, the area under the curve, likewise, has an area of 2(*a* + *b*). Consequently, the box describes how many artifacts we can expect to detect as though we found all of them within W/2 of the transect line, when in reality we found 2*b* artifacts within W/2 and 2*a* artifacts outside W/2. What this means, and makes sweep width useful and intuitively attractive, is that we expect to find the same number of artifacts as we would if we had perfect detection within the sweep width (Fig. 2). Keep in mind that our sweep width and the exponential detection function on which it is based only apply to the artifacts that are potentially visible on the surface, when we are using fieldwalking and visual inspection as our search method. We would have a very different sweep width if we were employing some other method, such as magnetic survey, augering, or test pitting, and wanted to consider buried artifacts, but in principal the sweep width measure is applicable to all these methods.

## Examples from Northern Jordan and Southwestern Cyprus

To demonstrate the operation and results of such calibrations, and their use in the planning and evaluation of actual surveys, here we use data from the Wadi Quseiba Survey of 2012–13 in northern Jordan and the 2014 Tremithos Valley Survey in southwestern Cyprus.

The main objective of the Wadi Quseiba Survey was to discover late prehistoric (Epipalaeolithic, Neolithic, and Chalcolithic) sites, which are commonly underrepresented in Jordanian surveys because of erosion, colluviation, the rarity and poor preservation of Neolithic pottery (cf. Bintliff et al. 1999), and sometimes the shortage of lithics expertise on survey teams. In an attempt to maximize the recovery of such sites, we employed a Bayesian algorithm for allocating search effort. Shortage of space precludes detailed discussion of this approach here, but suffice to say that it involves allocating new search effort on the basis of a predictive model that is continually updated in light of information on results, including coverage, as survey progresses (Hitchings et al. 2013).

The Tremithos Neolithic Survey Project is a pedestrian survey of sections of the Tremithos River Valley, running southeast from the Troodos foothills to the sea, in south-central Cyprus (Stewart n.d.). The goal of the project is to identify early Neolithic use of the valley to access the resource-rich areas in the Troodos foothills, particularly their abundant and high-quality chert sources, and whether this river system provided a transportation route from the sea to this central area. As in the Wadi Quseiba Survey, the Tremithos Neolithic Survey Project has employed Bayesian optimal allocation of survey effort, making it critical to estimate coverage and to update these estimates daily.

## Calibration Methods and Results

**Fig. 3:**
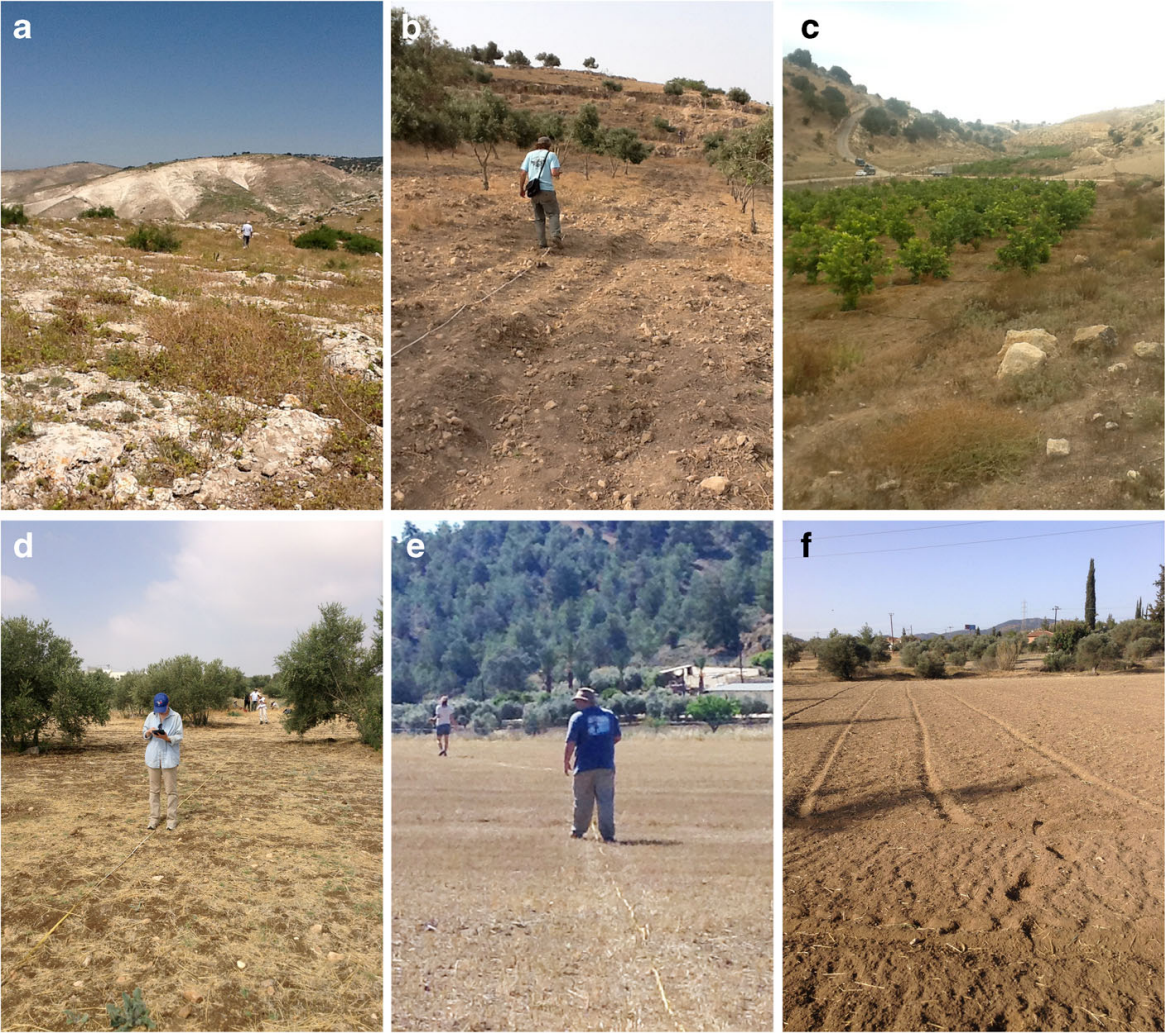
Views of the calibration fields **a** pasture, **b** olive grove, **c** guava orchard, **d** mixed field, all in Jordan, **e** stubble field, and **f** plowed field in Cyprus

In contrast to our previous simulations that employed a grid of strings as the test fields for “seeded” artifacts (Banning et al. 2006, 2011), to calibrate these surveys we experimented with test fields without a formal grid so as to be more similar to actual survey conditions, but that appeared to be devoid of ancient artifacts. In Jordan, test fields were set up in pasture that had a mixture of bare rock and patchy vegetation, a plowed guava field, a plowed olive grove, and a “mixed” field that had nearly equal portions of plowed field with some stubble and sparse trees, pasture with denser trees, and pasture with bare rock and sparse shrubs (Fig. 3a–d). In Cyprus, the test fields included a bare, plowed field and a field with wheat stubble (Fig. 3e–f).

In each test field, we laid out 50-m tapes to mark the path of a transect 120 or 150 m long, in some cases needing to bend this transect once or twice to avoid obstacles or allow for the shape of the field, and planted artifacts at locations randomly assigned to various locations left and right of the tape at distances up to 10 m (in the case of the “pasture”) or 20 m. Artifacts included the categories of large and small lithic flakes and large and small ceramic sherds in several colors, mostly “red” and “yellow” and somewhat similar to those anticipated in survey. However, for the purposes of this paper, we group the artifacts into all lithics and all pottery, whose sizes range from about 4 to 12 cm in greatest length. Initially, the calibrations also included very small lithics (microliths), but surveyors never detected them and we exclude them from the examples here.

Each member of the survey team walked along the tape multiple times over the course of several hours on each occasion that we conducted a calibration. Using a form on an iPad, on each attempt the surveyor marked his or her start and finish times and listed (but did not collect) each artifact seen along with its artifact category, distance along the 50 m tape, and, because there was no grid, estimated distance to left or right.

We kept the density of seeded artifacts low enough for us to be reasonably certain of our identification of “successful” detections (as opposed to “false targets”), even given the imprecision with which surveyors sometimes estimated the distances to artifacts they saw. Generally, these estimates were accurate and precise at close range, but we allowed for errors of up to 2 m in any direction at ranges of 15 to 20 m, and 1 m in any direction between 5 and 15 m.

**Fig. 4:**
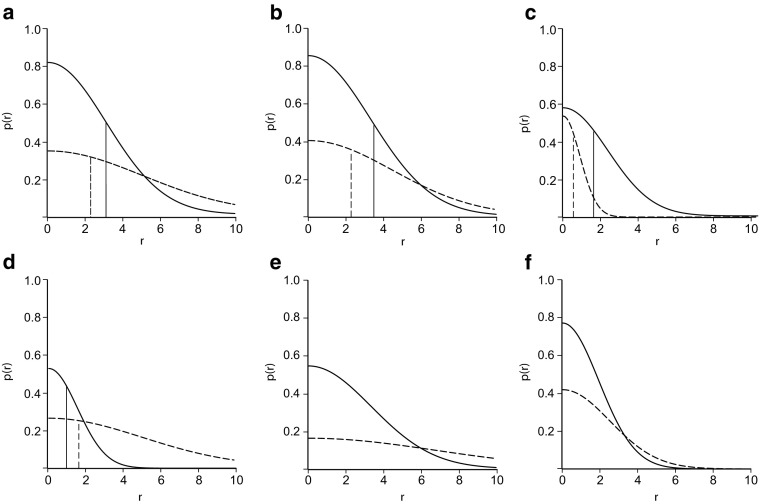
Detection functions for all lithics (*solid curve*) and all pottery (*dashed curve*) along with half the corresponding sweep widths (*vertical lines*) at the calibration sites **a** pasture, **b** olive grove, **c** guava orchard, **d** mixed field, all in Jordan, **e** stubble field, and **f** plowed field in Cyprus. p(*r*) is “probability at range *r*” and *r* is range in meters

By checking the data for all the transects and all crew members against the known locations of seeded artifacts, we are able to tabulate the number of successful detections by range and use the data to fit to a curve for the detection function (1) using nonlinear regression in either the SPSS^™^ or R platform to provide robust estimates of *b* and *k* using guesses of their values at the beginning of a constrained (0 ≤ *b* ≤ 1.0) model with estimation by sequential quadratic programming. We then use a numerical integration function in R to plot curves with these values and estimate the areas under the curves between ranges of −100 and 100 m to obtain sweep widths (Figs. 4 and 2).

As the curves and Table 2 make clear, there is considerable variation with type of field. Although small sample size may be a factor, the “best” sweep widths are in the pasture and the plowed olive grove in Jordan, where the sweep widths for lithics are on the order of 6–7 m and those for pottery are more than 4 m. The guava orchard, despite also being plowed, has noticeably poorer visibility. This probably has less to do with the plowing (although plowing was coarser, with deeper furrows than in the olive grove) than with the spacing and leafiness of the trees, which were young and had low branches.

**Table 2:** Estimated values for *b* and *k* in the fitted detection functions, along with standard deviations (±) and sweep widths (W) in meters for calibrations in four test fields in Jordan and two in Cyprus

	Field	*n*	Lithics					Pottery				
			*b*	±	*k*	±	W	*b*	±	*k*	±	W
Jordan	Pasture	12	.82	.09	.053	.012	6.3	.373	.032	.02	.004	4.7
	Olive Grove	17	.856	.089	.046	.010	7.0	.411	.018	.025	.002	4.6
	Orchard	67	.581	.069	.095	.023	3.3	.539	.192	.671	.317	1.2
	Mixed Field	45	.532	.072	.216	.055	2.0	.264	.09	.02	.02	3.3
Cyprus	Stubble	11	.163	.162	.011	.030	2.7	.550	.099	.045	.017	4.6
	Plowed Field	20	.751	.133	.135	.048	3.6	.422	.165	.079	.070	2.7

*n* represents the number of distinct traverses of the test field

The very low value of *b* for lithics on the stubble field in Cyprus and the depressed shape of the curve close to the y-axis compared with that for lithics in other locations can best be explained by our calibration methods. We chose to randomize locations of seeded artifacts, and, in this case, no lithics were seeded within 2 m of the transect and the resulting curve was based on artifacts observed at distances of 3 m and more. Consequently, we recommend, rather than using a completely random distribution, to use a uniform distribution with range and only randomize with distance along the transect.

We should not generalize too much from these results, since of course they depend in part on the abilities of the particular crew members involved, and not just on visibility or artifact obtrusiveness. We should also consider these somewhat optimistic estimates, since the artifacts were fresh and clean, rather than half-buried or dirty, and a few crew members appear to have walked a bit more slowly than they would normally do. However, they did provide us with some empirical data with which to assess our survey effectiveness, beginning with very preliminary estimates of sweep width (after only day of calibration) and leading up to the estimates provided here. Since the sweep widths for different artifact types differ, we were “conservative” by using the lesser of the lithic or pottery one. In addition, because visibility conditions in our actual surveys could only be approximately matched with the test fields, we adjusted our estimates slightly upward or downward from the estimates derived from the most similar test field, again attempting to be conservative by risking under-estimate of sweep width rather than over-estimate. However, because we have photographs of every transect segment in our database, it remains possible to gauge these estimates with image analysis at some later date, a method we will leave to another paper.

## Assessing Coverage for Optimal Allocation of Survey Effort

**Fig. 5:**
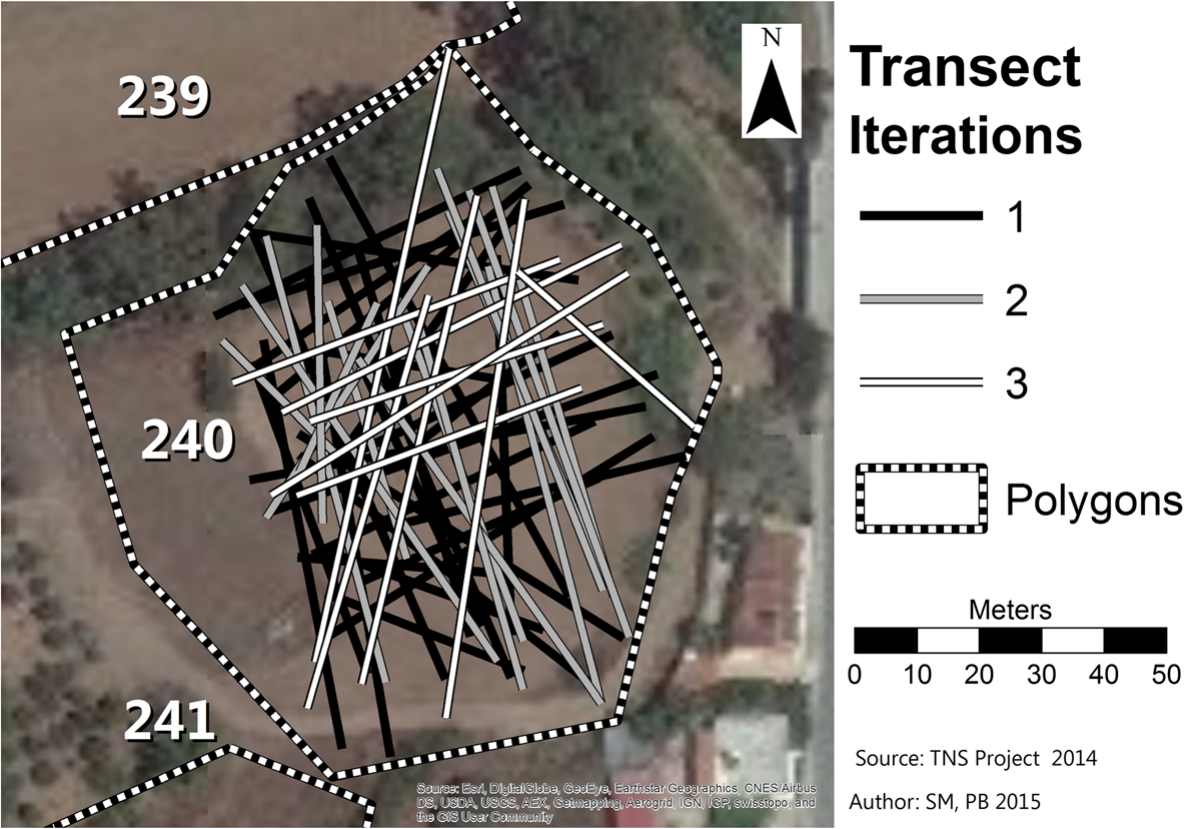
Map of multiple transects within polygon no. 240 in Cyprus. Note that new increments of survey use different transect orientations than previous iterations in order to maximize coverage of new ground and allow viewing of the ground from different angles

During the surveys in both Jordan and Cyprus, we used the sweep widths in conjunction with other information to help us allocate our survey effort each day through an iteratively updated predictive model. GPS coordinates for the beginnings and ends of transects walked allowed us to estimate the lengths of transects. Multiplying these lengths by our estimated sweep widths yields the total area “swept” by our transects in each survey unit. Dividing area swept by the total area of the survey unit tells us the coverage of that unit. This estimate of coverage is the key for us to reassess the probability that any survey unit (or “polygon” in our usage) might still contain undetected resources of interest. Because the kinds of sites that interest us tend to have very low artifact densities that make clustering not very evident, we did not explicitly take clustering into account but at each iteration of the predictive model recalculated the probability that each polygon might contain an undetected site of interest *given the total amount of coverage to date*. Thus, when we surveyed a polygon without finding identifiable late prehistoric materials, the polygon’s probability went down, but not necessarily by very much. Applying our allocation algorithm on the next iteration of the predictive model, it might easily lead us to survey that polygon again, and, indeed, we surveyed some polygons several times before finding anything of interest (Fig. 5; Hitchings et al. 2013). Note that in these instances we varied the orientation of transects to allow viewing of the ground from different angles and reduce redundancy in coverage (Banning 2002: 90–91; Koopman 1980: 218).

## Using Coverage to Demonstrate Exhaustion

**Fig. 6:**
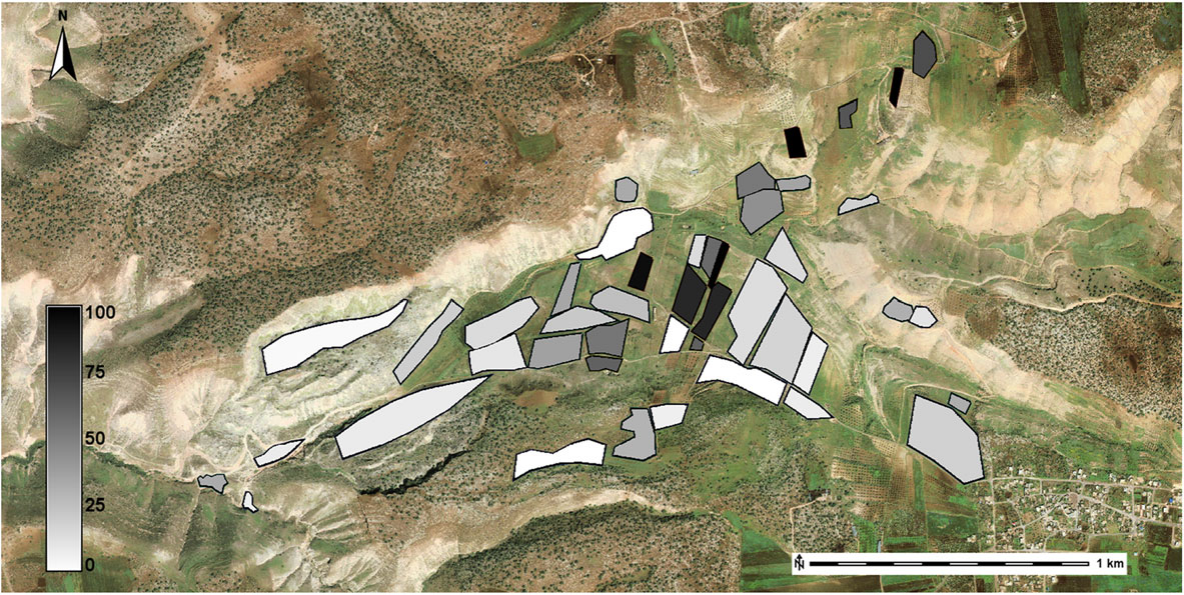
Exhaustion map for sub-region 2 in the Wadi Quseiba Survey, Jordan, indicating our estimated total coverage using conservative estimates of sweep width. The underlying imagery (*unshaded*) from DigitalGlobe has no formal coverage

In addition, the final coverage estimates provide our final assessment of how thorough our surveys were in both Wadi Quseiba and the Tremithos Valley, allowing other researchers to assess which parts of these survey regions, if any, might still repay further fieldwork. One way to express the overall quality of the survey’s coverage is to plot the coverage values on an “exhaustion map,” which displays our final estimate of how thoroughly each space in the survey region has been searched (Fig. 6). Exhaustion, for the purposes of this map, expresses the probability that there could still be undetected archaeological resources in a particular space, given the total amount of coverage that the survey has completed.

Exhaustion maps are particularly useful when a survey has only covered a portion of the survey region. When, for example, the survey has only intensively examined a sample of the region’s spaces, it is important to show exactly where survey took place and where it did not, so as not to give the misleading impression that unsurveyed spaces are devoid of archaeological materials. Reports should also provide justification for differential allocation of search effort, and sometimes tests to ensure that these differences did not bias the results in unintended ways.

**Fig. 7:**
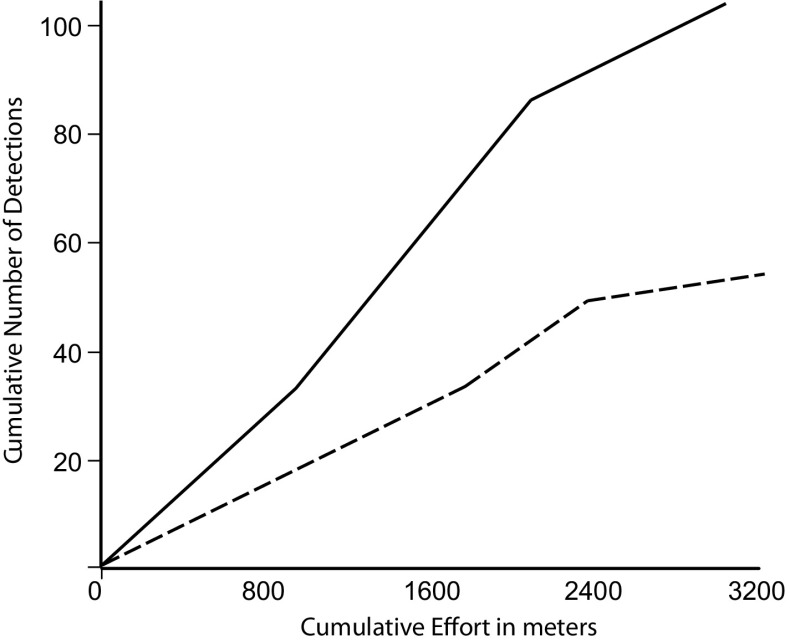
Graph of the cumulative number of artifacts detected with increments of survey effort in polygon 229 (*solid broken line*) and 240 (*dashed broken line*) in Cyprus. Note how the recovery of artifact levels off with increasing effort as measured in total meters walked

For particular spaces in the survey region, another approach for assessing survey thoroughness is to determine whether the detection function has begun to level off with increases in search effort. Using the examples of polygons 229 and 240 in the Tremithos Valley survey, we can see how the cumulative numbers of lithic finds increase over three increments of survey effort (Fig. 7). This graph would likely level off sooner had we been collecting or flagging all artifacts as they were found, but we only collected a sample, so some of the artifacts found in the second and third increments of survey could be ones that the first transects already identified. Even so, once this curve begins to level off, it is usually more effective to move onto a different space that has not received as much survey effort (Banning et al. 2006).

## Design Assurance in Archaeological Survey

Not only do the tests described in the previous section allow us to assess the quality of present and past surveys, they also provide information with which we can better design future ones.

## Transect Intervals

An excellent example of this is our ability to use the range functions to help us select appropriate transect intervals. The range functions allow us to estimate, within error margins, what transect interval is necessary to result in recovery of some proportion of the artifacts exposed in each transect. Effective visibility (E), for example, is the conventional term for half the interval that we would expect to result in detection of 50 % of the targets by parallel transects (Koopman 1980: 76). In some circumstances, finding half the artifacts might be adequate for achieving the goals of a research project or heritage assessment. For example, blue transfer ware might be found in high enough densities at typical nineteenth-century farmsteads in southern Ontario that only finding half of them is still adequate for the identification of these farms. In others, however, we might require better recovery than this and could calculate the appropriate interval for discovery of perhaps 80 % of the artifacts exposed on the surface or even use a transect spacing of W, which has an expected yield of 100 % of the artifacts. In still others, even 50 % might be a hopelessly unrealistic or costly goal, and we might have to settle for, say 20 %. Furthermore, the recovery of different artifact types varies, requiring us to prioritize their use in deciding transect intervals. In any of these cases, we can assure clients or academic audiences that we have met some predetermined level of artifact recovery, which constitutes the standard for the project or assessment firm.

## Crew Assignments and Training

Members of field crews can vary substantially in their overall detection abilities as well as in which artifact types they are most effective at detecting (Bintliff et al. 1999: 153; Stewart 2006: 140–144). This has a number of implications for our use of these personnel in future surveys, and, although here we have only considered the combined or average abilities of crews, it is possible to use our methods to assess individual performance as well.

In some cases, repeated testing might show that some crew members, despite further training, simply do not have the knack for artifact detection during fieldwalking. It might therefore make sense to reassign them to other tasks, such as test pitting, excavation, or lab work, where their talents are more suited.

In most cases, however, the tests will identify weaknesses in surveyors’ detection of certain artifact types that training or relatively simple changes in behavior can mitigate. Increasing their familiarity with artifact types that they tend to overlook, perhaps by having them spend some time in processing or helping to analyse them in the lab, is one way to improve their recognition of these types. Repeated practice survey on a test field seeded with this artifact type, or on an archaeological scatter that includes a good deal of this type of material, will also likely improve their detection of more difficult artifact types. In a few cases, something as simple as updating a prescription for corrective lenses or removing sunglasses may make the necessary difference.

Even after training and repeated testing, it is still likely that some surveyors will be better at detecting, say, lithics, while others are better at detecting nails or pottery. This has important implications for the mix of surveyors on field crews. Doubtless, a crew whose members complement one another’s strengths and weaknesses will have greater overall success at detecting a range of materials and site types than one that simply reinforces one kind of strength at the expense of others (Hawkins et al. 2003; Stewart 2006: 144–147). Consequently, survey managers should make crew assignments that tend to maximize the overall probability of detecting a range of artifact types that are likely to be important to the survey’s goals.

In the Wadi Quseiba and Tremithos Valley surveys, we only used sweep widths for the aggregate data of entire field crews, providing an average assessment of survey quality that is adequate for estimating overall survey coverage by those crews. This allowed us to have a reasonable sample size with which to fit the detection functions, although the raw data do indeed show considerable variation among surveyors. Where the composition of field crews varies substantially over the course of a survey, or there are other reasons to measure individuals’ sweep widths, it is necessary to ensure an adequate sample size of calibration transects for each surveyor.

## Conclusions

The reputability and professionalism of archaeological surveys depend, in part, on their ability to convince us that spaces in which they report a lack of archaeological materials are actually devoid of such materials. They also depend on the ability to characterize the existing materials correctly in terms of their type, density, and distribution. Consequently, ensuring the quality of archaeological surveys requires both careful attention to design and periodic testing of surveyors and survey procedures to determine their effectiveness and to strive for improvement.

Periodic assessments of crews’ effectiveness at artifact detection, using real artifacts under fairly realistic conditions, provide the data with which to accomplish these goals. In some regions, these assessments would need to be repeated in different seasons or be calibrated to recent rainfall amounts, but the key is to conduct them under conditions that are as close as possible to those of the actual survey. In surveys in northern Jordan and central Cyprus, where it virtually never rains during seasons when surveys typically occur, our calibration transects were across spaces with realistic field conditions. We had seeded these with modern artifacts similar to the kinds of artifacts we expected to find, which allowed us to estimate average sweep widths for the field crews. Multiplying the transect lengths by these sweep widths allows us to calculate total area swept in each survey space or “polygon,” and dividing this by the polygon’s area provides a reliable measure of coverage. These coverage estimates themselves provide a measure of survey thoroughness and thus one of the most important aspects of quality in survey. Using these coverage estimates in a Bayesian modeling environment further allows us to update the probabilities that each space in our survey frame might contain undiscovered archaeological materials, which we use both for allocating further survey effort and as another measure of the thoroughness of survey.

The result, we argue, provides a much stronger empirical basis for inferences about the distributions and character of regional archaeological resources.
